# Bridging the Digital-Analog Gap in Later Life: Testing the Interrelation of Internet Use and Social Participation

**DOI:** 10.1093/geroni/igaf122.1330

**Published:** 2025-12-31

**Authors:** Nicole Memmer, Anna Schlomann, Hans-Werner Wahl

**Affiliations:** Heidelberg University, Heidelberg, Baden-Wurttemberg, Germany; Network Aging Research, Heidelberg, Baden-Wurttemberg, Germany; Heidelberg University, Heidelberg, Baden-Wurttemberg, Germany

## Abstract

We target the question whether Internet use enables or impedes social participation in older adults’ analog world. Doing so, we test a rather comprehensive model that includes multiple predictors (i.e., cognitive attitude, affective attitude, and stereotype endorsement) of Internet use, different types of Internet use (i.e., social use, information seeking, entertainment), and a multi-indicator assessment of social participation, encompassing leisure activities as well as perceived social support and loneliness. Data come from the baseline assessment of the randomized controlled SMART-AGE trial. The analytical sample consists of 649 community-dwelling adults aged 67–93 (mean = 75.8; female = 52%). Structural equation modeling was applied to estimate the association of predictors with different types of Internet use and the relation of these usage patterns with social participation. Additionally, the study examines the moderating effect of digital skill level and socio-structural meso-context, i.e., two cities in southwestern Germany, on these relationships. Results show that stereotype endorsement and cognitive attitude were significantly related to different types of Internet use. Social Internet use exhibited the strongest positive association with social participation, while the path from information seeking to social participation was weaker, and entertainment revealed a null effect. Individuals with higher digital skills were found to derive greater social benefits from Internet use. Multi-group comparison revealed a weak but statistically meaningful impact of socio-structural meso-context on the inter-relations among Internet use and social participation. We conclude that a differential perspective is needed to understand the link between Internet use and social participation.

